# Ternary cyclodextrin systems of terbinafine hydrochloride inclusion complexes: Solventless preparation, solid-state, and in vitro characterization

**DOI:** 10.1016/j.heliyon.2023.e21416

**Published:** 2023-10-21

**Authors:** Balázs Attila Kondoros, Dávid Kókai, Katalin Burián, Milena Sorrenti, Laura Catenacci, Ildikó Csóka, Rita Ambrus

**Affiliations:** a Institute of Pharmaceutical Technology and Regulatory Affairs, University of Szeged, H-6720, Szeged, Hungary; b Albert Szent-Györgyi Health Center, Department of Medical Microbiology, Faculty of Medicine, University of Szeged, H-6725, Szeged, Hungary; c Department of Drug Sciences, University of Pavia, 27100, Pavia, Italy

**Keywords:** Grinding, Mechanochemical activation, Solvent-free green technology, Polyvinyl pyrrolidone, Hydroxypropyl methylcellulose, Sulfobutyl-ether-beta-cyclodextrin

## Abstract

Cyclodextrins (CD) are used extensively in the pharmaceutical industry to improve the water solubility and bioavailability of drugs. Preparing ternary systems by applying a third component can enhance these beneficial effects. The complexation methods of these ternary systems are the same as those of two-component complexes. These methods are solvent (co-evaporation, co-precipitation, etc.) or solventless “green” techniques (co-grinding, microwave irradiation, etc.). Using solvent-free methods is considered to be an economically and environmentally desirable technology.

This study aimed to prepare ternary systems by the co-grinding method and evaluate the effect of a third component by comparing it to products obtained by solvent methods, binary systems, and marketed products. For that, we used terbinafine hydrochloride as a model drug, sulfobutyl-ether-beta-cyclodextrin as a complexation agent and 5 or 15 w/w% of polyvinylpyrrolidone K-90 (PVP) or hydroxypropyl methylcellulose (HPMC) as auxiliary components.

Physicochemical evaluation (X-Ray Diffractometry, Differential Scanning Calorimetry, Thermogravimetry) showed that new solid phases were formed, while Scanning Electron Microscopy was performed to study morphological aspects of the products. Fourier transform infrared spectroscopic measurements suggested different intermolecular interactions depending on the type of polymer. In vitro dissolution studies showed beneficial effects of CD and further improvement with the applied polymers. Products showed less cell toxicity with one exception.

Both polymers enhanced the physicochemical and in vitro properties, suggesting a greater bioavailability of the model drug. However, the percentage of polymers applied did not appear to be an influencing factor for these properties.

## Graphical abstract

**Figure:**
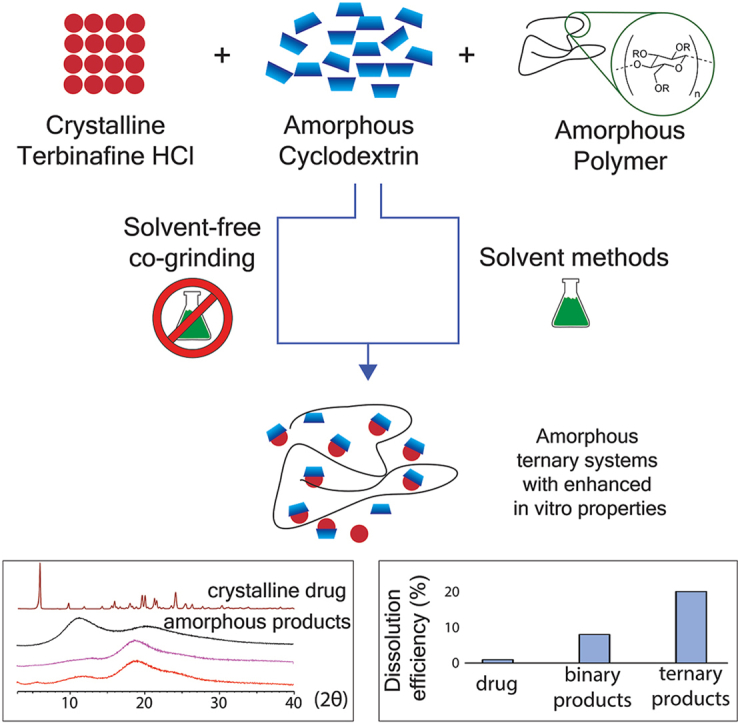

## 1 Introduction

This work concerns preparing and characterizing ternary systems of cyclodextrin (CD) and polymers with the allylamine antifungal drug Terbinafine hydrochloride (TER) as a model drug. This compound shows activity against yeasts, fungi, molds, and dermatophytes and is indicated for both oral and topical treatment of mycoses [1,2]. As one of the most effective drugs in the fight against dermatophyte fungi, it is included in the WHO's Model List of Essential Medicines [3]. Because of rapid absorption, TER reaches the maximum blood level about 1–2 h after administration [4] and it is widely distributed to body tissues, including the poorly perfused nail matrix, with a tendency to accumulate in the skin, nails, and adipose tissues [5].

The term CD refers to cyclic oligosaccharides, consisting of units of α-d-glucopyranose, bound by α-1,4 glycoside bonds [6]. The glucopyranoside units adopt a chair-like configuration, imparting CDs with a distinctive truncated cone structure characterized by two openings with varying diameters. Hydroxyl groups extend outward from the molecule, with primary and secondary hydroxyl groups positioned toward the wider and narrower edges, respectively, contributing to the hydrophilic nature of the exterior of the CD molecule. Conversely, the interior cavity of the CD is lined with carbon atoms and oxygen atoms from the ether linkages, resulting in a somewhat hydrophobic character. This duality of hydrophilic and hydrophobic characters gives amphiphilic properties to these molecules and makes CDs perfect for hosting hydrophobic molecules and creating water-soluble complexes [[7], [8], [9], [10]]. With complexation, hydrophobic drugs can improve their physicochemical properties and be used for various administration routes [[11], [12], [13]].

In pharmaceutical applications, binary systems are formed by the active ingredient with CD and ternary CD complexes represent supramolecular systems formed by the combination of three diverse materials [14]. The third component is often hydroxy acids, polymers, and amino acids and represents serving as a supplementary component that, when combined with CDs, further augments the intended physicochemical, chemical, and transport attributes of a specific drug [15]. These effects improve drug administration and reduce the amount of API and/or CD required in a given formulation, thus optimizing the expense, minimizing toxicity, and refining the size of the ultimate dosage form [16,17]. Among these materials, polymers are used most widely, such as chitosan, hydroxypropyl methylcellulose (HPMC), polyvinylpyrrolidone (PVP), polyethylene glycol, etc. [[18], [19], [20], [21]]. Incorporating hydrophilic polymers into binary complex formulations enhances the wettability of drug particles and facilitates the formation of easily soluble complexes. Polymers also can decrease the surface tension of water and enhance the hydration of CDs by reducing their mobility. Consequently, this process enhances the solubility of the complexes. Numerous research studies have demonstrated that these ternary systems exhibit improved in vitro solubility properties when compared to both polymer-containing and CD binary products, suggesting the presence of a synergistic effect [22,23].

Conventional preparation methods like co-evaporation, co-precipitation, and freeze-drying require a certain amount of solvent [24]. However, the purchase, storage, disposal of the organic solvent, and control of the residual solvent content of the final product can be time, material, and energy-consuming [25]. Moreover, the absence of solvents reduces unwanted reagent consumption (e.g., via hydrolysis). Based on these disadvantages, pharmaceutical industries, as many other industries, prefer organic solvent-free preparation methods [26]. These “green” technologies also playing an increasingly important role in the production of CD complexes using microwave irradiation, sonochemistry, photochemistry and mechanochemistry [27].

According to the well-known approach, grinding is a powerful tool for reducing particle size [28]. On the other hand, the process of mechanical grinding can induce mechanochemical activation [29]. Mechanochemistry as a term means that different mechanical forces (typically friction, impact, collision, grinding) induce chemical reactivity [30]. Grinding two or more materials together, called co-grinding (CG), can change intermolecular interactions between components, leading to a new solid form with modified properties [27]. Manual grinding (by mortar and pestle) or using ball, oscillating or vibration mills are the tools in the laboratory to induce mechanochemical transformation [[31], [32], [33]]. It is regarded as one of the most straightforward approaches to form CD inclusion complexes in a solid-state. Moreover, due to its independence from organic solvents, it is seen as an economically and environmentally favorable technology [27].

This study aims to prepare ternary systems of a Biopharmaceutical Classification System (BCS) class II drug with a CD derivative and a polymer as a third component. As a continuation of the previous work, we used TER as a model drug [34]. Numerous research paper have demonstrated the potential of CD complexation to enhance the stability and solubility of TER, presenting a viable strategy for formulating this medication [35,36]. Nonetheless, the development of solvent-free methods for producing the API CD complex with polymers remains to be achieved. Sulfobutylether-beta-cyclodextrin (SBEBCD), an amorphous CD derivative was chosen for complexing agent. It is used widely in pharmaceutical industry, as it is accepted by both European Medical Agency and Federal Drug Agency. It is proven to be a great CD derivative for several drug, and for several administration routes, however there is no marketed per os product worldwide. PVP and HPMC were selected on the basis of their successful use in previous work [22]. In previous works, a polymer content of 15 % has been suitable for cyclodextrin products [37]. In this work, 5 % was used in order to test whether a lower content would be sufficient to improve the properties of the active ingredient. Each product was obtained by preparing a physical mixture (PM) in a 1:1 M ratio of CD:API and then adding 5 or 15 % w/w of polymer content. The solvent evaporation method was carried out by completely dissolving the materials in the selected solvent, while kneaded products required a minimal amount of solvent, and co-grinding was performed only by manually grinding the components. As a combination of these materials and methods, 12 products and their respective PM were prepared and evaluated by physicochemical and in vitro characterization. Measurements were also evaluated and compared with those of the TER-containing commercial tablet and the TER:SBEBCD binary system to investigate the beneficial effects of the polymers used. Finally, the solubility properties of the products discussed in this study were compared with the previous work of our research group.

## 2 Materials and methods

### 2.1 Materials

Terbinafine hydrochloride (TER): (E)-N, 6,6-trimethyl-N-(naphthalene-1-ylmethyl) hept-2-en-4-yn-1-amine hydrochloride was provided by Gedeon Richter Plc. (Budapest, Hungary). SBEBCD(DS∼2) was provided by Sanofi-Aventis Ltd. (Paris, France). Hydroxypropyl methylcellulose (Methocel F4M; HPMC; apparent viscosity in water: 3000–5600 cP) was kindly supplied by Colorcon (Dartford, UK). Polyvinylpyrrolidone (PVP): K-90, molecular weight: 1300000. As a reference, a commercially available tablet containing TER was used per os tablet (Terbisil™ 250 mg, Gedeon Richter Plc., Budapest, Hungary) was used.

The preparation of simulated intestinal fluid (SIF) and simulated gastric fluid (SGF) without enzymes were conducted relyingon Chapter 5.17.1 of the European Pharmacopeia (10th edition). To prepare SIF (pH 6.8), a mixture was created by combining 77.0 mL of 0.2 M NaOH, 250.0 mL of a water-based solution containing 6.8 g of KH_2_PO_4_, and 500 mL of purified water (PW). The pH was then adjusted to pH 6.8, and the solution was further diluted to a total volume of 1000 mL using PW. For the preparation of SGF (pH 1.2), 1 g of NaCl was dissolved in PW, and 80 mL of 1 M HCl was added. Finally, the solution was diluted to a final volume of 500 mL with PW. In order to create 1 L of phosphate buffer solution (PBS), the following components were dissolved and then diluted to the desired volume of 1000 mL using PW: 1.44 g of disodium phosphate dihydrate (Na_2_HPO_4_ × 2H2O), 0.12 g of potassium dihydrogen phosphate (KH_2_PO_4_), 8.00 g of sodium chloride (NaCl), and 0.20 g of potassium chloride (KCl). All the materials utilized for these solutions were obtained from Sigma-Aldrich (Budapest, Hungary).

### 2.2 Methods

The TER:SBEBCD:PVP (TSP) and the TER:SBEBCD:HPMC (TSH) products obtained by different methods have been prepared from the same composition. The molar ratio of API to CD was 1:1 in all cases, and 5 or 15 %(w/w) of polymer (PVP or HPMC) was measured and added to make the physical mixture (PM). The solvent evaporation (SE) method was planned and carried out based on previous works [38,39], SE products were prepared by completely dissolving the physical mixture in 50 %(v/v) ethanol and evaporating the solvent at room temperature with continuous stirring, after that the product was dried at room temperature for 24 h in vacuum drying oven (Binder GmbH, Tuttlingen, Germany). In the case of kneaded products (KP) the same solvent was used but a minimal volume of it, and the products were kneaded in a mortar until the solvent evaporated. To prepare co-ground products (CG), PM were ground manually. After every 5 min samples were taken and characterized by X-Ray Powder Diffractometry (XRPD) to justify complete amorphization (results of this characterization can be seen in results and discussion section). As a result of these materials and preparation methods, 15 products were prepared (Table 1).

**Table 1:** Abbreviations based on materials and preparation methods for different products. Product codes contain letter T (TER) S (SBEBCD) and P (PVP) or H (HPMC), also preparation method (SE, KP, CG), and amount of applied polymer (0, 5 or 15 %).

No.	*Abbreviation*	*Methods*	*Materials*	*Percentage of polymer (%)*
**1**	**TS SE**	Solvent evaporation	TER, SBEBCD,	0
**2**	**TS KP**	Kneading		
**3**	**TS CG**	Co-grinding		
**4**	**TSP SE 5 %**	Solvent evaporation	TER, SBEBCD, PVP	5
**5**	**TSP SE 15 %**			15
**6**	**TSP KP 5 %**	Kneading		5
**7**	**TSP KP 15 %**			15
**8**	**TSP CG 5 %**	Co-grinding		5
**9**	**TSP CG 15 %**			15
**10**	**TSH SE 5 %**	Solvent evaporation	TER, SBEBCD, HPMC	5
**11**	**TSH SE 15 %**			15
**12**	**TSH KP 5 %**	Kneading		5
**13**	**TSH KP 15 %**			15
**14**	**TSH CG 5 %**	Co-grinding		5
**15**	**TSH CG 15 %**			15

To further analyze the crystallographic phenomenon, CG was re-performed with a Fritsch Planetary Micro Mill Pulverisette 7 premium line (Fritsch GmbH, Idar-Oberstein, Germany) with the same composition. The PM was placed in 45 mL stainless steel grinding bowls with grinding balls of the same material, the rotational speed of the main disk was 600 rpm. To avoid heating the material during grinding, the machine was paused for 1 min after each 1-min grinding phase, and every 2 min of effective grinding time samples were taken. With this method milled 5 % and 15 % polymer containing TSH and TSP products were prepared.

#### 2.2.1 Phase solubility study

The solubility and the ability of TER to form complexes with SBEBCD in an aqueous solution were assessed using phase solubility methods following the procedure outlined by Higuchi and Connors [40]. To conduct the evaluation, excess TER was introduced into separate solutions containing varying concentrations of CDs, ranging from 0 to 50 mM. These solutions were agitated at 25 °C for 24 h within sealed containers. Afterward, the samples were filtered through a syringe membrane filter with a pore size of 0.22 μm, and their analysis was carried out using a Unicam UV/VIS spectrometer (Thermo Fisher Scientific, Waltham, USA) at 284 nm in order to quantify the amount of solubilized TER.

The data obtained from these experiments were employed to create a phase solubility diagram, plotting the dissolved TER against the concentration of SBEBCD. From this diagram, the apparent stability constants (KC) of the complexes were determined using the Higuchi–Connors equation:

(1) $$K_{C}=\frac{slope}{S_{0}\left(1-slope\right)}$$

where S0 is the intrinsic solubility of TER.

#### 2.2.2 Differential Scanning Calorimetry (DSC)

Temperature and enthalpy values were measured on 2–5 mg of sample weighed each time in 40 μL aluminum crucibles with a two-hole lid. Measurements were taken with a Mettler STARe system (Mettler Toledo, Novate Milanese, MI, Italy) equipped with a DSC821e module and an Intracooler device (Julabo FT 900) for sub-ambient temperature analysis with the following settings: heating from 25 to 300 °C with 5 °C/min heating rate with nitrogen purge (50 L/h). The data was evaluated using the STAR^e^ System software.

#### 2.2.3 Thermal gravimetric analysis (TG)

TG measurement was carried out using a Mettler TA 4000 apparatus (Mettler Toledo, Novate Milanese, MI, Italy). This equipment was fitted with a TG 50 cell and utilized 8–10 mg samples placed in open alumina crucibles. The experiments were conducted with a heating rate of 5 K/min under a static air atmosphere, and the temperature range examined ranged from 25 to 300 °C.

#### 2.2.4 X-ray powder diffractometry (XRPD)

XRPD measurements were conducted using a Bruker D8 Advance diffractometer (Karlsruhe, Germany). These measurements utilized Cu-KαI radiation with a wavelength of 1.5406 Å. The X-ray tube operated at a voltage of 40 kV and a current of 40 mA. Diffractograms were recorded in the angular range of 3–40° (2θ) with a pitch of 0.007° and a time constant of 0.1 s. The obtained data were evaluated with the Bruker DiffracPlus Eva software.

#### 2.2.5 Fourier-transform infrared spectroscopy (FT-IR)

Spectra of raw materials, PM, and products were recorded by a Spectrum One Perkin–Elmer FTIR spectrophotometer (Perkin–Elmer, Wellesley, MA, US) equipped with a MIRacleTM ATR device (Pike Technologies, Madison, WI, US). The measurements were conducted within the spectral range of 650–4000 cm^−1^, employing a resolution of 4 cm^−1^. To achieve a high signal-to-noise ratio, 32 scans were performed and subsequently averaged. On every product, 3 parallel measurements were carried out to prevent deviation due to the possible inhomogeneity of the samples. The recorded spectra were subjected to similar adjustments, averaging the 3 parallel measurements and applying baseline correction and peak normalization by Spectragryph - optical spectroscopy software [41].

#### 2.2.6 Scanning electron microscopy (SEM)

The morphological characteristics of the samples were examined using scanning electron microscopy (SEM; Hitachi S4700, Hitachi Scientific Ltd., Tokyo, Japan) The SEM analysis was conducted at 10 kV. Prior to imaging, the samples were coated with a thin film of gold-palladium, approximately 10 nm in thickness, using a coater sputter (Bio-Rad SC 502, VG Microtech, Uckfield, UK). Only TER and samples containing 5 % (TSP 5 % and TSH 5 % products) of polymer were analyzed and presented. The particle size measurement results were obtained using ImageJ 1.44p software (Bethesda, MD, USA), particles were measured manually and evaluated by the software. Statistical analysis was carried out to assess whether there was a significant difference among the measured data. In the case of all the characterized products, a unidirectional variance analysis (ANOVA) was conducted, followed by the post hoc Tukey HSD test. Similarly, the particle diameters of the samples generated through various preparation methods were compared. Experimental outcomes with p-values less than 0.05 and 0.01 were considered to be statistically significant.

#### 2.2.7 In vitro dissolution rate studies

Dissolution rate studies of pure TER drug and prepared solid inclusion complexes of TSH, TSP, TS systems, and pulverized Terbisil™ tablet were carried out. According to European Pharmacopeia Dissolution studies were carried out using a dissolution apparatus employing the paddle method at 37 °C, with a rotational speed of 100 rpm, but with a reduced volume of the dissolution medium (50 mL). SIF and SGF were used as dissolution media. At specified time intervals (5, 10, 20, 30, 60, 90, and 120 min), 5 mL portions were withdrawn from the solution and simultaneously replaced with fresh dissolution medium. These withdrawn samples were promptly filtered through a syringe membrane filter with a pore size of 0.22 μm. After appropriate dilution with the dissolution medium, the concentration of the dissolved drug was quantified using a Unicam UV/VIS spectrometer from Thermo Fisher Scientific (Waltham, MA, USA) at a wavelength of 284 nm.

Cumulative dilution caused by the medium replaced during sampling has been considered. To quantify the dissolution curves, two metrics were used, dissolution efficiency (DE) and mean dissolution time (MDT), calculated according to the following equations. DE represents the area under the dissolution curve up to a specified time and it is expressed as a percentage of the rectangle area and can be calculated using the equation:

(2) $$\text{DE}=\frac{\int_{0}^{t}y\mathrm{d}t}{y_{100}}100\%$$

where y and y100 are the cumulative percentage dissolution at time t and 100 % dissolution, respectively [42]. MDT is used to characterize the drug release rate of TER and the products, using the following equation:

(3) $$MDT=\frac{\sum_{i=1}^{n}t_{mid}\Delta M}{\sum_{i=1}^{n}\Delta M}$$

where i is the dissolution sample number, n is the number of dissolution times, t_mid_ is the time at the midpoint between times t_i_ and t_i−1_ and ΔM is the amount of TER dissolved (mg) between the same intervals [42].

#### 2.2.8 Cytotoxicity studies

To assess cell viability data, the mitochondrial activity was evaluated using an MTT (3-(4,5-dimethylthiazol-2-yl)-2,5-diphenyltetrazolium bromide) assay in 96-well cell culture microplates employing Caco-2 cells (human colorectal adenocarcinoma). Caco-2 cells were initially seeded at a density of 4 × 104 cells per well. Compounds were serially diluted two-fold, with the highest TER concentration being 1 mg/mL and the lowest 0.001953 mg/mL. Subsequently, the samples were incubated at 37 °C for 24 h. Following this incubation, 20 μL of MTT reagent was added to each well. After an additional 4-h incubation at 37 °C, 100 μL of 10 % sodium dodecyl sulfate was introduced, and after a subsequent 12-h incubation, the optical density (OD) was measured. Cytotoxicity was determined by measuring the OD at 550 nm (with reference to 630 nm) using an EZ READ 400 ELISA reader from Biochrom in Cambridge, UK. The viability of untreated cells was considered 100 %, and the viability of the tested products was compared to this control. Each concentration was subjected to four replicate assays.

## 3 Results

### 3.1 Phase solubility study

**Fig. 1:**
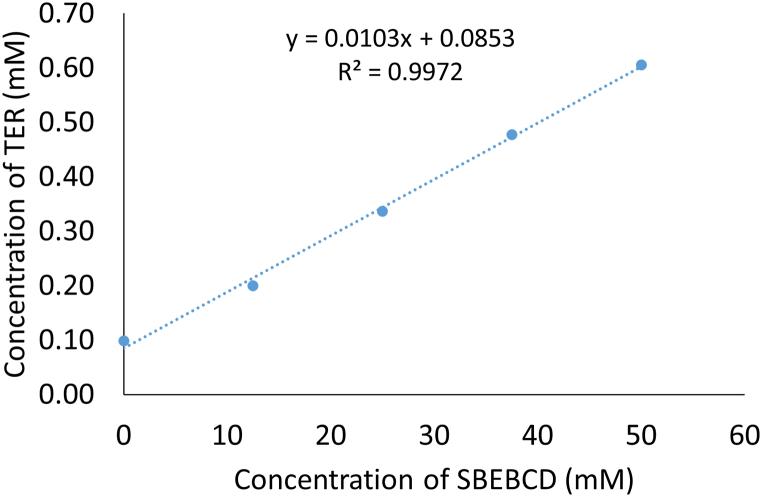
Phase solubility diagram of TER in aqueous solutions containing SBEBCD at 25 °C.

Fig. 1 shows the enhanced solubility of TER as the concentration of SBEBCD increases. The plotted diagram displays a straight line with slopes less than 1, suggesting characteristic of Higuchi A_L_ type. This indicate the formation of a 1:1 molecular ratio between the API and the CD. Using the Higuchi–Connors equation, the apparent solubility constant (K_C_) was determined from Fig. 1, resulting in a value of 122.01 M^-1^.

### 3.2 Thermoanalytical characterization

**Fig. 2:**
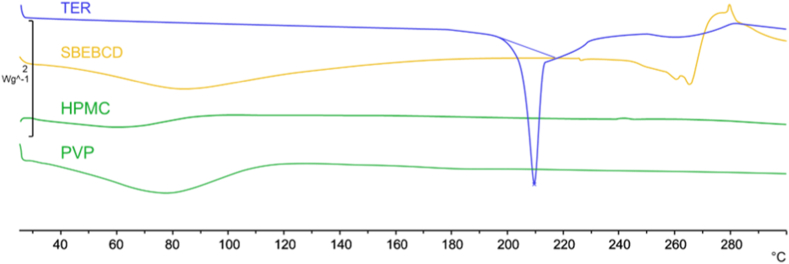
DSC thermograms of TER (blue), SBEBCD (yellow) and the two polymers HPMC and PVP (green).

The comparison of the thermal curves of the individual components, their PM, and the presumed inclusion complexes provides information on the changes of the solid-state and the interactions between the components, a consequence of how the various procedures used are decisive for the formation of the inclusion complex. The DSC data showed a well-defined peak related to TER at 209 ± 0.2 °C, followed by decomposition observed at higher temperatures (Fig. 2, blue curve). In the DSC curves obtained for the various samples, enlargements, and displacements of the melting peak can be observed, related to the partial loss of TER crystallinity, because of the solid-state interactions between the components.

The DSC curve of the SBEBCD (Fig. 2, yellow curve) showed the amorphous nature of the CD derivative, characterized by an intense and broad endothermic effect with a peak around 90–100 °C corresponding to dehydration corresponds to moisture present in the sample, followed by a decomposition above 280 °C. For PVP, a large endotherm ranging from 40 to 110 °C has been observed indicating water loss. A similar thermal profile with a wide endothermic transition at around 60 °C was recorded for HPMC (Fig. 1, green curves).

**Fig. 3:**
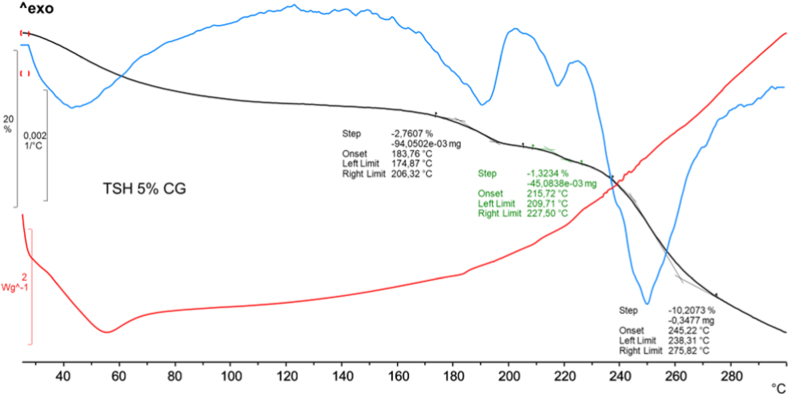
DSC (red), TG (black), 1st derivative of TG (blue) curves of TSH 5 % ground product.

TG measurements were conducted to delve deeper into the thermoanalytical characteristics of the materials and to better understand the decomposition observed in the DSC measurements. Every product showed a multistep mass loss, 1st derivative of the TG curves was calculated and reported along with TG and DSC curves for easier determination of each inflection point. In Fig. 3 are reported DSC, TG and DTG curves recorded on TSH CG 5 %, as representative of every prepared ternary system, all other products showed the same phenomena.

The original water content of the CD can be detected as weight losses at the beginning of the TG curves, in correspondence with the dehydration process, were observed for all products. The following mass decreases seen in the curves indicate the degradation of compounds, as confirmed by the prolonged complex phenomenon seen in the DSC curves. The complete disappearance of the melting point of the crystalline drug in the DSC curves of the alleged complexes can be considered as supporting evidence for the inclusion of the drug molecule within the CD cavity. Since TER and the CD decompose in a similar temperature range, the two phenomena cannot be distinguished from each other by TG curves. Comparing the TG curves of TER as pure drug with the ternary systems, it was also observed that the decomposition occurred in the same temperature range, suggesting that the products did not increase the thermal stability of the active substance.

### 3.3 X-ray powder diffractometry (XRPD)

To detect changes in solid-state properties as a result of the preparation methods and the interactions between components, XRPD patterns on powder of the individual components and the products were compared. The reduction and changes in their relative intensities of the distinctive peaks of the host molecule are factors that suggest the formation of a new solid phase and therefore support the effective formation of the inclusion complex. X-ray diffractogram of TER is characterized by sharp peaks, suggesting crystalline properties, while amorphous materials, such as CD and the polymers used, do not show well-defined peaks, confirming their non-crystalline nature.

Methods of preparation of the complexes by SE and CG gave rise to amorphous products with both polymers. However, the obtained KP products presented the characteristic peaks of TER, confirming the only partial loss of crystallinity. In general, the percentage of polymer used have no influence on the crystalline nature of the products obtained.

**Fig. 4:**
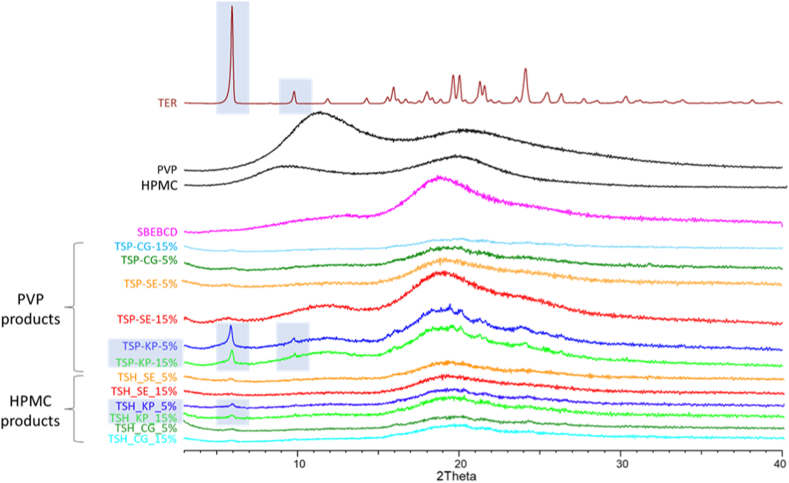
XRPD diffractograms of raw materials and all products. Crystalline peaks were highlighted in the case of TER and KPs.

Amorphization was promoted by the continuous supply of energy from grinding. A longer grinding time induced a greater loss of crystallinity. The characteristic peaks of the drug have decreased the intensity with increasing grinding time. In the case of the product containing 5 % PVP, the time required for a complete amorphization was 70 min of the grinding process, while all other products required a lower grinding time of 30 min. The same behavior was achieved for all the systems obtained, irrespective of the polymer used for the preparation of the ternary system (Fig. 4). The same grinding procedure was also applied for pure TER, for the longest time (70 min) used as for the ground products and did not produce the amorphization of the active ingredient.

However, these effects can only be conclusively linked to the transformation of the crystalline compound into an amorphous state. This transformation might be the result of the formation of a real inclusion complex, or it may also stem from various other processes that occurred during the sample preparation.

**Fig. 5:**
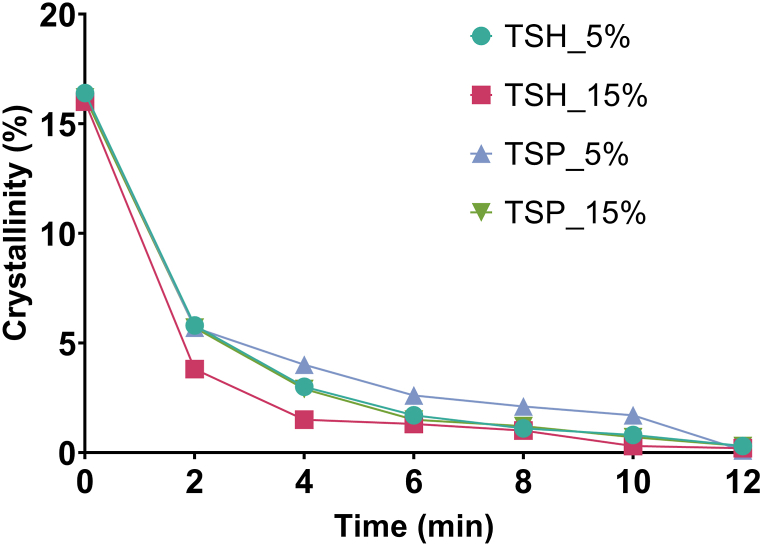
Percentage of the crystalline material of milled samples as a function of time.

To further investigate the crystallographic changes induced by grinding and to evaluate the effect of polymers on grinding time, mechanochemical activation was carried out using a milling machine with unchanged material composition. The initial degree of crystallinity was found to be about 16 %, as the PM also contained a large amount of amorphous material due to the presence of the excipients, which resulted in a large amorphous background on the diffractograms. Plotting crystallinity versus grinding time gave similar curves for all products. The steepest slope was experienced in the first 2 min, reaching the minimum crystallinity detectable by the instrument in 12 min (Fig. 5).

### 3.4 Fourier transform infrared spectroscopy (FTIR)

**Fig. 6:**
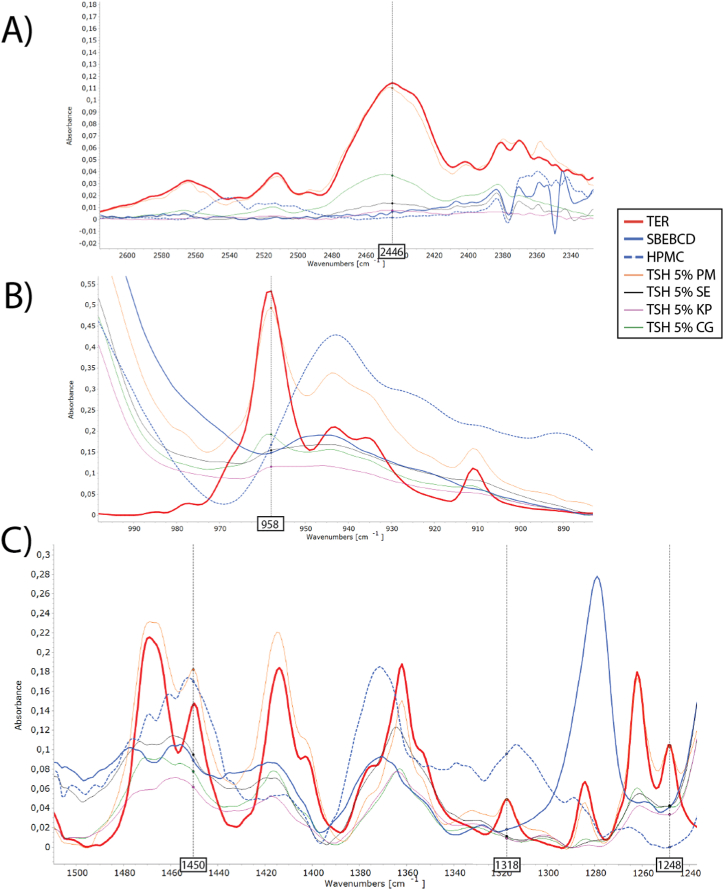
FTIR spectra of TSH products and corresponding raw materials and their PM.

The FTIR analysis provided additional information regarding the intermolecular interactions of TER and the excipients. TER spectrum contains characteristic intense bands at 2968, 2446, 1361, 1631, 1514, 958, 1450, 1318, and 1248, and three bands between 820 and 760 cm^−1^. Products resulted in similar spectral changes, for PVP and HPMC products, respectively. A broad brand of the amine group can be seen at 2446 cm^−1^, that disappears in SE and KP products but is detectable in CG products (Fig. 6A). For aromatic out of a plane (three bands between 820 and 760 cm^−1^) and aromatic C=C bond (1514 cm^−1^) there is no shifting or intensity decrease. The band corresponding to the aliphatic C=C group (1631 cm^−1^) is not detectable in the products due to the presence of a broad band characteristic of the CD in the same region. The *trans*-substituted olefin group band at 958 cm^−1^ was detected only in the CG products reduced and shifted (Fig. 6B). Other C=C bands due to stretching vibration at 1450, 1318, and 1248 cm^−1^ completely disappeared or in the case of CG products of HPMC significantly reduced and shifted (Fig. 6C). However, in the case of PVP, the band at 1318 cm^−1^ is not suitable for interpreting interactions because the PVP presented a large band in this spectral range.

There are some differences in spectral changes depending on the polymer used. For aromatic C–H deformation bands referring to the methyl and methylene groups at 2968 cm^−1^ disappearance of the peak can be seen in the spectra of TSP SE products and shifting in TSP KP and CG products, while there were no changes in the spectra of HPMC-products. Similarly, shifting of *t*-butyl axial deformation (1361 cm^−1^) can be detected in all PVP products, in HPMC KP and CG products no changes occurred, except for shifting bands of TSH SE products. Overall, the amount of polymers used did not affect the interactions that occurred, so for better transparency, only products containing 5 % of polymer are shown in Fig. 6.

Overall the FTIR measurements revealed that the different methods used to obtain ternary complexes and polymer excipients used resulted in different effects on the formation of non-covalent bonds between the drug and CD.

### 3.5 Scanning electron microscopy (SEM)

**Fig. 7:**
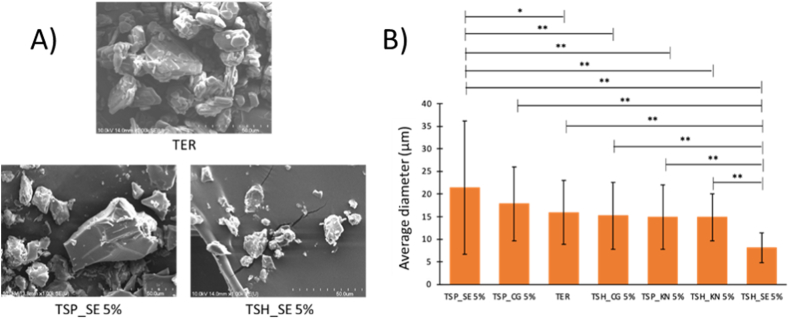
SEM images (A) of TER and SE products. Average diameter (B) of products containing 5 % of polymer. Experimental results with p-values less than 0.05 (*) and less than 0.01 (**) were considered to indicate statistical significance.

SEM was performed for TER and the products obtained with the various techniques, to investigate their morphology and particle size (presented in Fig. 7A and B respectively).

TER crystals appear as aggregates, showing a flat crystalline profile with an average diameter of 15.904 ± 7.090 μm. The surface of the crystals is smooth with rounded edges and free of cracks. SE produces smooth-surface samples with small portions of polymer film-like cohesive material to form a film in the case of HPMC. For samples containing PVP the sample electron micrograph showed larger crystals with smooth surfaces and sharp edges.

SE method gave very uncertain results in terms of particle size, the average diameter was 21.408 ± 14.791 μm for the sample obtained with PVP, and 8.122 ± 3.279 μm for the sample with HPMC. In this case, the larger size was achieved using PVP as a polymer, which showed a significant difference (p < 0.01) with all other samples except for TSP CG. There was also a significant difference (p < 0.05) in size with TER. Using HPMC polymer the particle size was significantly different (p < 0.01). So, the method of evaporation of the solvent is not desirable from the point of view of the processability of the product, because it is not possible to control its particle diameter and therefore is not recommended for the manufacture of these products.

Using the KP preparation method there was no significant difference in the size of the samples prepared with the two polymers. The particle size is 14,924 ± 7110 μm for PVP products, while for products with HPMC 14,869 ± 5224 μm. The SEM images also show a strong similarity, with particles having small, irregular, and flat morphology.

As with the KP, the size of the samples produced by CG did not depend on the polymer used. In fact, with both PVP and HPMC, CG produced particles with a size similar to that of untreated TER crystals. The particle size was 17,813 ± 8157 μm for products with PVP and 15,178 ± 7441 μm for products with HPMC. A key difference with the other samples is that the grinding process made the particles rough and wrinkled, rather than smooth. Also, in this case, the shape of the particles was irregular.

### 3.6 In vitro dissolution rate studies

**Fig. 8:**
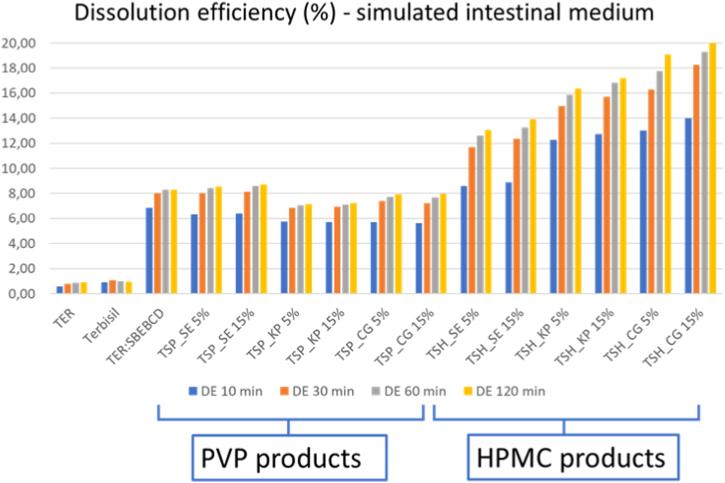
DE% in SIM during in vitro dissolution studies at 10, 30, 60, and 120 min.

In vitro dissolution studies were carried out to assess the dissolution characteristics of the formulations. Compared to the pure drug, the binary system, and the commercial product Terbisil®. The solubility of a drug, which is influenced by the pH of its environment, can result in incomplete dissolution or precipitation, ultimately affecting its bioavailability. This concern may be relevant for TER, particularly at higher administered doses. Different pH mediums were tested in vitro dissolution studies to get a complete characterization of the TER release profiles. For experiments performed in SIM dissolution efficiency (DE) was calculated at 10, 30, 60, and 120 min (Fig. 8). Pure drugs and Terbisil® showed less than 1 % DE which can increase to approximately 8 % with SBEBCD alone. PVP products did not increase it any higher, suggesting no effect of this polymer on solubility increase. However, HPMC products raised it to approximately twice, with differences between products. Among these, CG products were outstanding with the highest DE% (120 min) value, approximately 20 %.

**Fig. 9:**
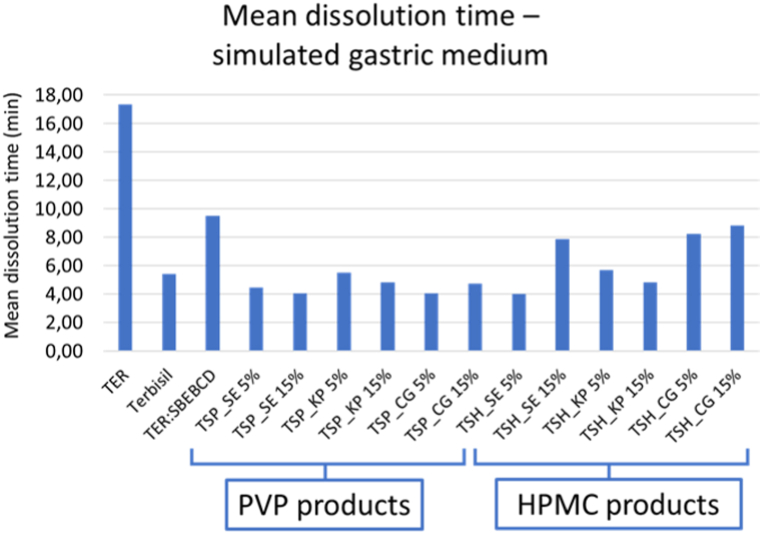
MDT values measured in SGM.

A lower pH, in the SGM dissolution of TER is higher. More than 90 % of the pure drug is dissolved after 60 min. However, products reach this level in a shorter time, so the dissolution rate is higher. To quantify this phenomenon, mean dissolution time (MDT) was calculated (Fig. 9). It shows a high dissolution time for TER and a lower one for each product and even for the marketed product. However, none of the products shows significantly lower MDT than the binary system.

One of the objectives of the present work is to compare SBEBCD containing products with previous results regarding HPBCD and DIMEB. Table 2 contains data of the binary systems and the ternary system with the highest DE value (TSH CG 15 %). This shows that the products prepared with binary SBEBCD had better leaching properties than DIMEB and HPBCD used in previous experiments [34]. Moreover, the addition of HPMC to the mixture also had a positive effect.

**Table 2:** Comparison of products of a previous and the present study. The DE (%) values of CG products at calculated from the 120 min dissolution study data.

	TER:HPBCD	TER:DIMEB	TER:SBEBCD	TSH CG 15 %
DE (%) 120 min	1.70 [34]	4.81 [34]	8.31	20.11

### 3.7 Cytotoxicity studies

**Fig. 10:**
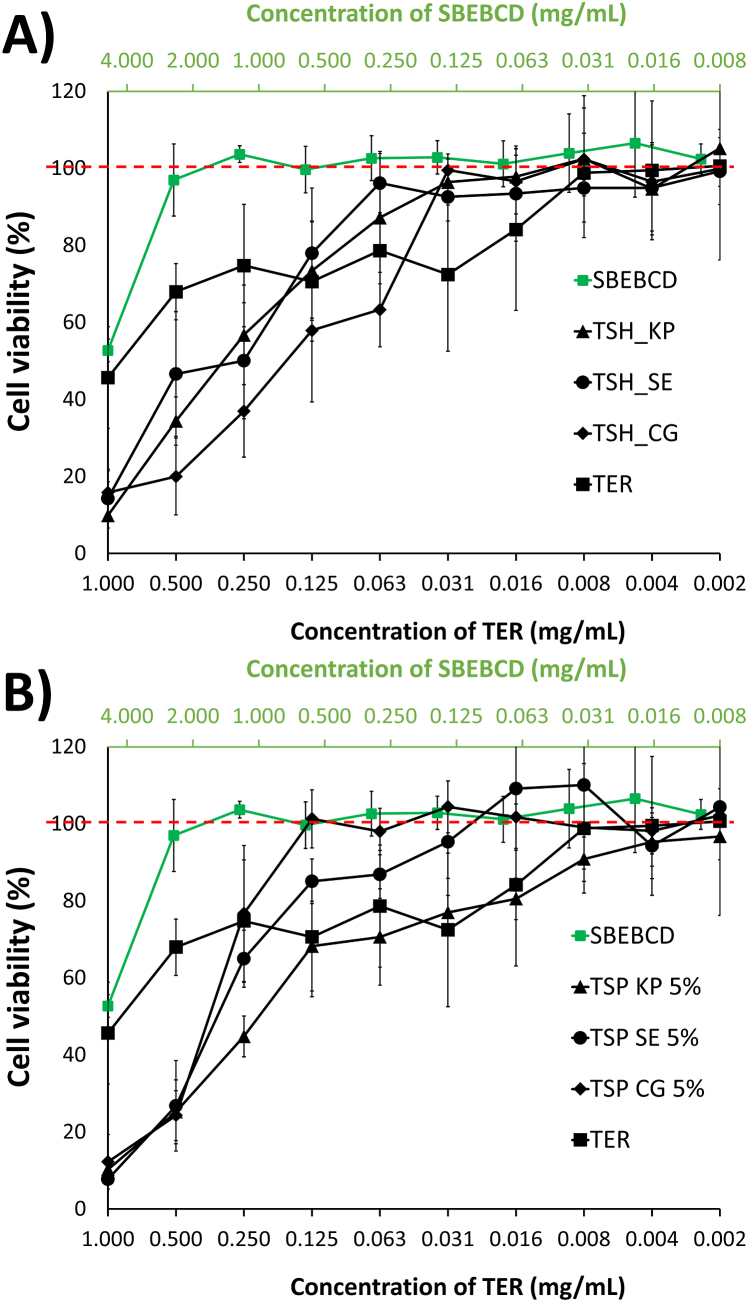
Cell viability (%) as a relationship between TER concentration (lower x-axis) and SBEBCD concentration (upper x-axis) in reverse order on a logarithmic two scale. The two raw materials are shown in both graphs. Products with PVP (A) and HPMC (B) showed in black, since those were depicted as their TER-content concentration. 100 % of cell viability was marked with red dashed line, as a marker of non-toxic level.

Based on the results of the solid phase and dissolution rate studies, which revealed no differences depending on the concentration of polymer used, cytotoxicity studies were only performed for samples containing 5 % of polymer (cell viability data shown for PVP and HPMC products in Fig. 10 A and B, respectively). MTT assay studies were performed to investigate the effect of the products and the active ingredient on cell viability. Pure TER, SBEBCD and ternary products were tested with this method. For TER and the products concentration was applied in all cases at a serial two-fold dilution of 1 mg/mL TER concentration. The concentration of the SBEBCD was at a serial two-fold dilution of 4.89 mg/mL (Table 3).

**Table 3:** Concentration of materials in the starting solutions for cytotoxicity measurements. All other samples were diluted from these starting solutions at a serial dilution. TER concentration was 1 mg/mL in the starting solution of pure TER and all products.

Sample	Concentration (mg/mL)			
	TER	SBEBCD	PVP	HPMC
SBEBCD	0.00	4.89	0.00	0.00
TER	1.00	0.00	0.00	0.00
TSP products	1.00	4.89	0.31	0.00
TSH products	1.00	4.89	0.00	0.31

The least cell toxic material among the tested samples was SBEBCD (marked with green in Fig. 10). Only the highest, 4.89 mg/mL concentration sample showed decreased cell viability, all other dilution were in non-toxic range.

TER showed 45.66 (±13.21) % cell viability at 1 mg/mL, and cell viability of all products was lower than that of pure TER at this concentration. However, when pure TER was diluted, cell viability remained in the toxic range until a dilution of 0.008 mg/mL. Products showed less cell toxicity, since they reached non-toxic level with higher TER concentrations (in the range of 0.125 and 0.31 mg/mL). The only exception was TSP KP 5 %, where the highest non-toxic concentration was 0.004 mg/mL, suggesting worst cell viability properties (Fig. 10).

## 4 Conclusion

The modifying effects of two different polymer excipients, PVP and HPMC on the physicochemical and in vitro properties of a TER-SBEBCD complex were investigated. Both polymers were used at two concentrations, 5 and 15 w/w%. These ternary complexes were compared with the binary complex, the drug alone and the commercialized formulation of TER in per os tablets.

Different intermolecular interactions – revealed by FTIR – could result in different thermoanalytical and in vitro behaviors. Overall, both excipients modified the properties of the complex in a positive direction, but no differences were observed between the percentage of polymer used to prepare the systems.

The 5 w/w% systems could provide the same synergic effect in vitro results as the 15 % products and did not change the trend of crystallinity decrease. Firstly, this means that a lower concentration of the polymer is enough to reach the same effect. On the other hand, the product with 15 w/w% of the polymer contained a lower concentration of CD, which raises an economic aspect. Namely, the relatively expensive CD derivative - and with it, the active ingredient in a 1:1 M ratio - can be reduced by adding a larger amount of relatively cheap polymer.

All of this suggests that the use of polymers can positively impact on bioavailability and should be taken into account in the design of a future product also considering the lower material cost of producing complexes.

## Data availibility statememnt

No data was used for the research described in the article.

## Additional information

No additional information is available for this paper.

## CRediT authorship contribution statement

**Balázs Attila Kondoros:** Investigation, Methodology, Writing – original draft. **Dávid Kókai:** Investigation. **Katalin Burián:** Conceptualization, Methodology. **Milena Sorrenti:** Investigation, Methodology. **Laura Catenacci:** Investigation. **Ildikó Csóka:** Supervision, Writing – review & editing. **Rita Ambrus:** Conceptualization, Investigation, Methodology, Project administration, Supervision, Writing – review & editing.

## Funding

The publication was funded by The 10.13039/501100015763University of Szeged Open Access Fund (FundRef, 554 Grant No. 6286).

## Declaration of competing interest

The authors declare that they have no known competing financial interests or personal relationships that could have appeared to influence the work reported in this paper.
